# Editorial: Impact of Climate Change on Poultry Metabolism

**DOI:** 10.3389/fvets.2021.654678

**Published:** 2021-02-26

**Authors:** Kyung-Woo Lee, Joris Michiels, Yang-Ho Choi

**Affiliations:** ^1^Department of Animal Science and Technology, Konkuk University, Seoul, South Korea; ^2^Department of Animal Sciences and Aquatic Ecology, Ghent University, Ghent, Belgium; ^3^Department of Animal Science and Institute of Agriculture and Life Sciences, Gyeongsang National University, Jinju, South Korea

**Keywords:** heat stress, chicken, metabolism, climate change, performance

Climate change is a major environment-origin challenge for the global animal industry as it adversely affects performance and health of animals, particularly chicken. Chickens are vulnerable to heat stress and they can only tolerate narrow ranges of temperature for maximum productivity. This collection of six papers (four research papers and two reviews) focuses on the recent insight of how heat stress affects poultry.

Kang et al. studied the effect of a rapid vs. gradual increase in temperature on the physiology of laying hens. They identified that a rapid elevation of temperature had more severe effects on mortality in hens. The results from the study of Kang et al. provide clear evidence of the inability of laying hens to tolerate thermal environments in which temperature was rapidly elevated to high levels. The importance of incorporating reliable tools to slow or monitor temperature rises within the poultry house is addressed.

Liang et al. reviewed the sprinkler technology for broilers as a physical intervention to mitigate heat stress within the poultry house. The technology utilizes direct sprinkling on the surface (i.e., feathers) of poultry with coarse water droplets, thus accelerating evaporation to dissipate sensible heat. Liang et al. emphasized that sprinkler cooling uses 66% less water than used in a conventional evaporative cooling system (i.e., fogging). Of interest, the authors found that sprinkler technology exhibited beneficial effects on cellular metabolism by preventing heat stress-induced AMPK activation, hence preserving intracellular levels of ATP. It is considered a promising technique to conserve water usage and to improve performance and well-being of poultry.

Kim et al. investigated the effect of heat stress on the performance and physiology of laying hens exposed to various stresses conditioned from low (22°C), moderate (27°C), and high (32°C) temperatures. Hens from the high temperature group had the highest rectal temperature, heart rate, and infra-red recorded surface temperature at all time points measured. In addition, high temperatures had poor laying performance and eggshell qualities via lowered feed intake coupled with alterations in gut microbiota and mineral metabolism. However, these authors found that heterophil to lymphocyte ratio and corticosterone in plasma and yolk cannot be considered a reliable indicator for the level of heat stress. Rather, they propose that body-surface temperature measurement by intra-red thermography is a reliable quantitative stress indicator.

Gut health of chickens is important as it orchestrates performance via enhanced digestion and absorption processes. Impaired gut integrity can increase susceptibility to enteric diseases such as those caused by *Eimeria* spp. or *Clostridium perfringens*. Tabler et al. investigated the fundamental mechanisms associated with the effect of heat stress and broiler strain on intestinal barrier function. For this purpose, the authors selected multiple biomarkers including blood FITC-D concentrations, and intestinal expression of heat shock and tight junction proteins in broilers. Tabler et al. highlighted that tight junction protein expression is environmental-, genotype-, and intestinal segment-dependent. They identified molecular signatures, such as cadherin 1, calprotectin, and zonula occludins 1, potentially involved in leaky gut syndrome as induced by heat stress in fast-growing broilers compared with slow-growing counterparts.

Chowdhury et al. reviewed recent developments of heat stress relieving nutritional strategies for chickens with a particular focus on citrulline and leucine. The interest in these nutrients is from the findings that heat stress decreases plasma citrulline in chickens and leucine in the embryonic brain and liver. Indeed, Chowdhury et al. emphasized that dietary citrulline lowered body temperature of layer chicks exposed to heat stress and *in ovo* leucine feeding attenuated heat stress-induced decrease in body weight of broiler chicks. Chowdhury et al. proposed that incorporation of amino acid (i.e., leucine and citrulline) into the diets could be a novel nutritional approach in assisting poultry to cope with heat stress.

The observation that early exposure to heat stress has been known to increase the tolerance of chickens to heat stress led Ouchi et al. to investigate the physiological and molecular changes of thermogenesis related molecules in the liver and muscle of chicks. Early thermal conditioning increased the expression of myosin and actin genes indicating activation of protein synthesis and lowered rectal temperature. Metabolomic and gene expression analysis revealed that early thermal conditioning increased the level of hepatic arginosuccinic acid and related enzymes (i.e., argininosuccinate synthase and argininosuccinate lyase) affecting heat production. In addition, thermal conditioning lowered the gene expression of avian uncoupling protein and carnitine palmitoyltransferase-1 in pectoral muscle indicating a decrease in heat production. Ouchi et al. observed that thermal conditioning can reduce heat production in muscles of chickens and may promote energy trapping process in the liver by altering the heat production system.

The papers gathered in this Research Topic provide an update on the impact of heat stress *per se* on nutrition, physiology, and health of chickens. Furthermore, recent development on heat stress relieving strategies including nutrition-based *in ovo* technology, direct water sprinkling and early thermal conditioning was introduced. This Research Topic will contribute to help the global poultry industry to mitigate the heat stress leading to improving health and welfare of chickens.

## Author Contributions

All authors listed have made a substantial, direct and intellectual contribution to the work, and approved it for publication.

## Conflict of Interest

The authors declare that the research was conducted in the absence of any commercial or financial relationships that could be construed as a potential conflict of interest.

